# Resolvin E1 as a Potential Biomarker of Tendon Retraction Severity in Rotator Cuff Tears

**DOI:** 10.3390/jcm14248887

**Published:** 2025-12-16

**Authors:** Recep Taskin, Sedat Gülten, Mehmet Akif Bildirici, Osman Sabri Kesbiç

**Affiliations:** 1 Department of Orthopaedics and Traumatology, Faculty of Medicine, Kastamonu University, 37150 Kastamonu, Türkiye; 2 Department of Biochemistry, Faculty of Medicine, Kastamonu University, 37150 Kastamonu, Türkiye; sgulten@kastamonu.edu.tr (S.G.); mabildirici@kastamonu.edu.tr (M.A.B.); 3 Department of Animal Husbandry and Nutrition, Faculty of Veterinary Medicine, Kastamonu University, 37150 Kastamonu, Türkiye; okesbic@kastamonu.edu.tr

**Keywords:** Resolvin E1, tendon retraction, rotator cuff tear, inflammation resolution, biochemical biomarker

## Abstract

**Background/Objectives**: Specialized pro-resolving lipid mediators (SPMs), such as Resolvin E1 (RvE1) and Resolvin D1 (RvD1), play a critical role in the resolution phase of inflammation. However, their relevance to tendon pathology and tissue-specific degeneration in rotator cuff tears remains unclear. This study aimed to investigate the relation between serum RvE1 and RvD1 levels and the morphological severity of tendon retraction and muscle fatty degeneration in patients with full-thickness rotator cuff tears. **Methods**: A total of 70 participants were included: 35 patients with full-thickness rotator cuff tears determined by magnetic resonance imaging (MRI) and 35 healthy controls. Tendon retraction and muscle fatty degeneration were graded using Patte and Goutallier classifications, respectively. Serum RvE1 and RvD1 levels were measured using enzyme-linked immunosorbent assay (ELISA). Group comparisons were performed using Welch’s *t*-test, and correlations were analyzed with Spearman’s coefficient. **Results**: RvE1 and RvD1 levels were significantly lower in patients compared to controls (*p* < 0.001). RvE1 showed a moderate positive correlation with Patte score (ρ = 0.37, *p* = 0.027), while no significant correlation was observed with Goutallier classification (ρ = 0.19, *p* = 0.27). RvD1 levels demonstrated no significant relationship with either morphological parameter. **Conclusions**: These findings suggest that decreased serum RvE1 levels are associated with the severity of tendon retraction but not with muscle fatty degeneration. Therefore, RvE1 may serve as a potential biochemical biomarker reflecting tendon damage severity and the impaired resolution of inflammation in rotator cuff tears.

## 1. Introduction

Rotator cuff tears are common musculoskeletal disorders resulting from the loss of integrity of the muscles and tendons that provide stability to the shoulder joint due to degenerative or traumatic causes [1]. In the pathophysiology of these tears, not only mechanical stress but also chronic inflammation and the accompanying inadequate tissue healing play an important role [2]. Prolonged inflammatory processes lead to the accumulation of proinflammatory cytokines in the tissue microenvironment and, consequently, to irreversible structural changes such as fatty degeneration and tendon retraction [3].

In recent years, it has been understood that not only the initiation but also the resolution phase of inflammation is a decisive process in tissue repair [4]. This phase is regulated by bioactive lipids known as specific pro-resolving lipid mediators (SPMs). The SPM family includes resolvins, protectins, and maresins derived from omega-3 (n-3) polyunsaturated fatty acids, as well as lipoxins derived from omega-6 (n-6) arachidonic acid. These mediators perform key roles in the resolution of inflammation, such as terminating neutrophil infiltration, clearing apoptotic cells, and restoring tissue homeostasis [4,5]. Among these compounds, resolvins represent the best-characterized subgroup of SPMs, particularly because they are derivatives of EPA (eicosapentaenoic acid) and DHA (docosahexaenoic acid). Resolvin E1 (RvE1), derived from EPA, and Resolvin D1 (RvD1), derived from DHA, are leading members of this class of mediators that play an active role in the resolution phase of inflammation [4,6]. These lipid mediators reduce neutrophil infiltration, enhance macrophage phagocytic activity, and suppress the release of pro-inflammatory cytokines, thereby facilitating the physiological resolution of inflammation [7]. The course of the inflammatory response is influenced not only by biochemical processes but also by intrinsic and extrinsic factors. Intrinsic characteristics such as age, tendon vascularity, biomechanical quality of the tissue, and cellular regeneration capacity; extrinsic factors such as repetitive mechanical loading, impingement, metabolic syndrome, and systemic inflammatory conditions can determine the resolution capacity of inflammation. Changes in these factors can directly affect the levels of pro-resolving lipid mediators and the risk of inflammation becoming chronic. The acute inflammatory phase is characterized by rapid neutrophil infiltration, pro-inflammatory cytokine release, and increased vascular permeability in response to tissue damage. If this phase cannot be physiologically terminated, inflammation becomes chronic, leading to impaired macrophage polarization, continuous cytokine release, and reduced tissue regeneration capacity. A decrease or loss of function of pro-resolving lipid mediators facilitates the transition of inflammation from the acute to the chronic phase and contributes to the structural changes seen in tendon retraction and muscle degeneration. Therefore, RvE1 and RvD1 levels can be considered a biochemical indicator of inflammation resolution capacity [4]. The amount of EPA and DHA obtained through diet is considered a determinant of circulating RvE1 and RvD1 levels. Diets rich in n-3 fatty acids, particularly the regular consumption of fish and seafood, accelerate the resolution of inflammatory processes by supporting the biosynthesis of these resolvins [8,9]. Aquatic-based foods contain much higher amounts of long-chain n-3 fatty acids compared to terrestrial sources. Cold-water species such as salmon (*Salmo salar*), mackerel (*Scomber scombrus*) and sardines (*Sardina pilchardus*) are among the richest sources of EPA and DHA; therefore, regular consumption of these fish can significantly increase serum RvE1 and RvD1 levels [10,11]. Furthermore, it has been reported that optimizing the feed formulations of farmed salmonid species can preserve omega-3 concentrations in muscle tissue, creating a sustainable source for both human nutrition and inflammation resolution capacity [12]. Therefore, diets rich in n-3 fatty acids and fish consumption are considered a critical factor in supporting tissue repair by increasing the biosynthesis of resolvins, which are effective in the resolution phase of inflammation.

The roles of RvE1 and RvD1 in the resolution phase of inflammation offer a new perspective on understanding the biochemical basis of fatty infiltration and fibrotic remodeling processes caused by chronic inflammation in tendon and muscle tissues [13,14]. Chronic inflammation results in myofibrillar degeneration, increased adipogenic cell differentiation, and elevated fibroblastic activity in the rotator cuff muscles, thereby reducing the muscle’s contractile capacity [3,7]. In this process, specific pro-resolving lipid mediators such as RvE1 and RvD1 inhibit myofibroblast activation and reduce collagen accumulation by suppressing the NF-κB, MAPK, and TGF-β1/Smad signaling pathways [15]. RvE1 binds to the ChemR23 receptor, reducing the activation of pro-inflammatory macrophages (M1) while supporting the polarization of anti-inflammatory phenotype macrophages (M2) [16]. This change accelerates the transition to the resolution phase in muscle tissue and contributes to the prevention of fatty acid accumulation. Similarly, RvD1 has been shown to exert effects via the ALX/FPR2 and GPR32 receptors, suppressing lipid peroxidation and inhibiting the settlement of adipose progenitor cells in muscle tissue [4]. In experimental models, RvD1 administration has been reported to reduce intramuscular fat infiltration after muscle injury, stimulate the renewal of myosatellite cells, and significantly reduce the degree of fibrosis [17]. Furthermore, RvE1 has been found to support mechanical integrity by stimulating tenocyte proliferation and type I collagen synthesis in tendon tissue and reducing the expression of inflammatory cytokines such as TNF-α and IL-1β [13,15]. Therefore, RvE1 and RvD1 deficiency not only prolongs inflammation but also predisposes to lipid infiltration and structural weakening in the muscle-tendon complex. The decrease in tissue levels of these mediators may reflect an inadequate resolution response, consistent with the high Goutallier and Patte scores observed in rotator cuff tears.

There are a limited number of clinical studies in the literature examining the role of resolvins in musculoskeletal system pathologies, and existing research has mostly focused on inflammation resolution mechanisms in experimental models. In particular, there are few clinical studies evaluating the relationship between RvE1 and RvD1 and tendon retraction and muscle degeneration in rotator cuff diseases. This situation points to a significant knowledge gap in understanding the role of the resolution phase of inflammation in tendon pathobiology. This study is one of the clinical investigations evaluating the relationship between RvE1 and RvD1 levels and tendon and muscle morphology. It aims to make an innovative contribution to the literature by revealing the biomarker potential of pro-resolving mediators in rotator cuff tears.

## 2. Materials and Methods

### 2.1. Study Design

This study was designed as an analytical cross-sectional study to evaluate the relationship between serum RvE1 and RvD1 levels and tendon retraction and muscle fat in patients with full-thickness rotator cuff tears. Participants were included in the study using consecutive sampling. The sample size was calculated based on an effect size (d = 0.8) aimed at detecting the mean difference between two independent groups, and it was determined that a minimum of 26 participants were required for 80% power (power = 0.80) and a 5% significance level (α = 0.05).

Written informed consent was obtained from all participants and the study was approved by the Kastamonu University Non-Interventional Clinical Research Ethics Committee (Decision No: 38/2025).

### 2.2. Clinical Participants

This study included 35 patients who presented to the Orthopedics and Traumatology Clinic of Kastamonu University Education and Research Hospital 2025 and were diagnosed with a full-thickness rotator cuff tear by magnetic resonance imaging (MRI), as well as 35 healthy person who has applied to the hospital for a check-up with no clinical or radiological shoulder pathology. Both groups exhibited similar demographic characteristics in terms of age, gender, and side (Table 1).

The mean age of the patient group was 63.1 ± 10.4 years, while that of the healthy group was 52.3 ± 8.8 years. The patient group consisted of 22 women and 13 men, while the healthy group consisted of 20 women and 15 men. When examining the distribution of affected sides, rotator cuff tears were found in the right shoulder in 68.6% (*n* = 24) of patients and in the left shoulder in 31.4% (*n* = 11).

### 2.3. Imaging and Morphological Assessment

All patients’ shoulder magnetic resonance imaging (MRI) scans were performed using a GE Healthcare SIGNA™ Victor 1.5 T (Chicago, IL, USA) device at a magnetic field strength of 1.5 Tesla. Images were acquired in the coronal oblique, sagittal oblique, and axial planes. The sequences used included T1-weighted spin-echo, proton density (PD)-weighted fat-suppressed (PD-FS), and T2-weighted fat-suppressed (T2-FS) sections. The imaging parameters were optimized according to the institution’s standard shoulder protocol: slice thickness: 3.5 mm, image matrix: 256 × 256, field of view (FOV): 16 cm, and TR/TE values were T1-SE: ~500/12 ms, PD-FS: ~2500/45 ms, and T2-FS: ~4000/90 ms, optimized according to the institution’s standard shoulder protocol.

The degree of fatty degeneration in the rotator cuff muscles was determined by scoring it on a scale of 0–4 according to the Goutallier classification [18]: Stage 0: normal muscle, Stage 1: mild fatty streaks, Stage 2: <50% fatty infiltration of muscle tissue, Stage 3: approximately 50% fat and muscle mixture, Stage 4: >50% fatty tissue predominance.

Tendon retraction was assessed according to the Patte classification [19]: Stage 1: tendon ends at the level of the humeral head, Stage 2: tendon ends medial to the humeral head, Stage 3: tendon ends retracted to the glenoid cavity.

### 2.4. Blood Sampling and Measurement of RvE1 and RvD1 Levels

A 3 mL venous blood sample was collected from each participant into serum separator tubes. The collected samples were allowed to clot for 30 min, then centrifuged at 3000 rpm for 10 min to separate the serum fraction. The obtained serum samples were stored at −20 °C until the day of analysis. Sunred brand commercial ELISA kits (Shanghai Sunred Biological Technology Co., Ltd., Shanghai, China) were used for the quantitative determination of serum Resolvin E1 (RvE1) and Resolvin D1 (RvD1) levels. The analyses were performed according to the manufacturer’s instructions. Optical density values were measured at 450 nm wavelength using a microplate reader (Biotek 50 TS microplate washer and BioTek 800TS Microplate reader, Winooski, VT, USA) data collection and analysis were performed using KC Junior software (Biotex Instruments, Winooski, VT, USA).

### 2.5. Statistical Analysis

The statistical analysis of the data was performed using IBM SPSS Statistics for Windows (IBM Corp., Armonk, NY, USA) software, version 26.0. Continuous variables were expressed as mean ± standard deviation (SD), while categorical data were expressed as frequency (n) and percentage (%). The normality of the data distribution was assessed using the Shapiro–Wilk test. The independent samples *t*-test (Welch-corrected) was applied to compare age, serum Resolvin E1 (RvE1) and Resolvin D1 (RvD1) levels between groups. The distributions of categorical variables (gender, affected side) were assessed using chi-square (χ^2^) and binomial tests. The relationships between serum RvE1 and RvD1 levels and Goutallier and Patte classification scores in patients with rotator cuff tears were investigated using Spearman correlation analysis. The correlation coefficients (ρ) obtained were interpreted in terms of the direction and strength of the relationship. Additionally, Spearman correlation analyses were performed between age and serum RvE1/RvD1 levels to assess potential confounding effects of age. All statistical tests were performed using two-tailed significance, and *p* < 0.05 was considered statistically significant. ChatGPT (GPT-4o, OpenAI, 2025) was used for purposes such as improving the clarity of scientific writing and assisting in the visualization of certain metabolic pathways. 

## 3. Results

Serum Resolvin E1 (RvE1) and Resolvin D1 (RvD1) levels in the patient and healthy groups are shown in Table 2. Serum RvE1 level was determined to be 82.18 ± 12.87 pg/mL in the patient group and 142.37 ± 97.05 pg/mL in healthy individuals. Similarly, the RvD1 level was determined to be 81.63 ± 14.73 pg/mL in the patient group and 144.54 ± 98.55 pg/mL in the healthy group. The independent samples *t*-test (Welch’s corrected) performed for both markers showed that the values in the patient group were statistically significantly lower than those in healthy individuals (t = −3.64, *p* = 0.0009; t = −3.73, *p* = 0.0007 for RvD1). The effect size (Hedges g) values calculated for both markers were 0.84 (RvE1) and 0.86 (RvD1), respectively, indicating that this difference represents a large effect. Spearman’s correlation analysis revealed a strong negative association between age and serum RvE1 levels (r = –0.42, *p* = 0.001). A notable inverse correlation was identified between age and RvD1 levels (r = –0.41, *p* = 0.001). The findings suggest a decline in resolvin levels with increasing age.

These findings indicate that pro-resolving lipid mediators, which regulate the resolution phase of inflammation in patients with rotator cuff tears, are reduced and that the resolution capacity is impaired.

The relationship between serum Resolvin E1 (RvE1) and Resolvin D1 (RvD1) levels and Goutallier (muscle fatty degeneration) and Patte (tendon retraction) scores in the patient group was evaluated using Spearman correlation analysis (Table 3).

A weak but statistically insignificant positive correlation was found between RvE1 levels and the Goutallier score (ρ = 0.19, *p* = 0.27). In contrast, RvE1 levels showed a moderate positive and significant correlation with the Patte retraction score (ρ = 0.37, *p* = 0.027).

RvD1 levels did not show a significant relationship with either the Goutallier (ρ = 0.13, *p* = 0.46) or Patte (ρ = 0.27, *p* = 0.12) scores.

## 4. Discussion

This study demonstrated that serum RvE1 and RvD1 levels were significantly reduced in patients with full-thickness rotator cuff tears (*p* < 0.05), and that RvE1 in particular showed a significant correlation with the severity of tendon retraction. In contrast, no significant correlation was found between RvD1 levels and tendon or muscle morphology. Furthermore, it was determined that intramuscular fatty degeneration (Goutallier) is not associated with resolvin levels. These findings suggest that disruption in the resolution phase of inflammation may play a more prominent role, particularly in tendon structure.

**Figure 1:**
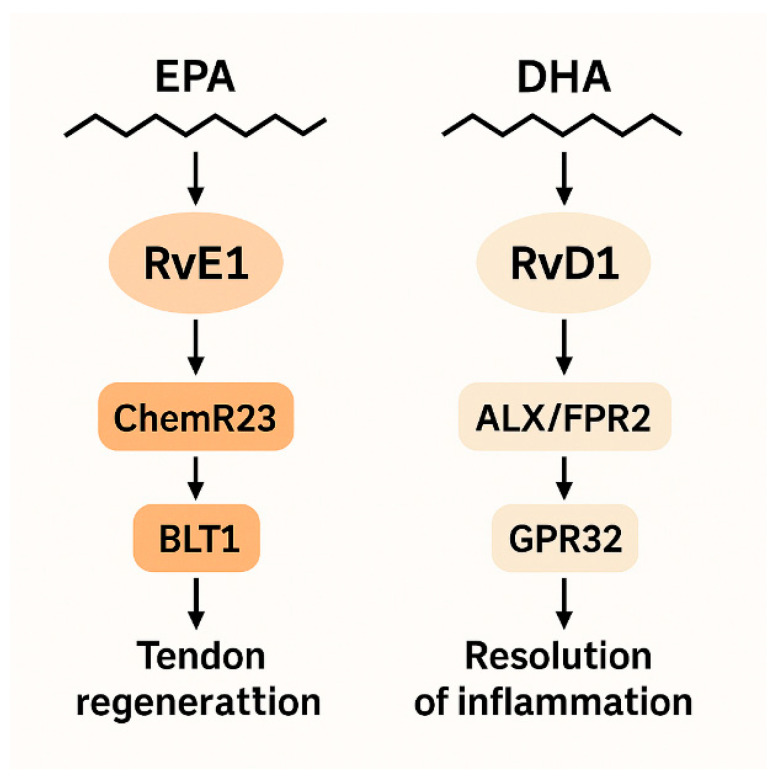
Receptor interactions of RvE1 and RvD1 and different signaling pathways during the dissolution phase.

Although RvE1 and RvD1 belong to the same biochemical class, their tissue-specific effect profiles differ [4,16]. RvE1, derived from EPA, acts via ChemR23 and BLT1 receptors, whereas RvD1, derived from DHA, functions through ALX/FPR2 and GPR32 receptors [5,6] (Figure 1). Collectively, these receptor differences may explain why RvE1 shows tendon-specific associations in our results.

Previous studies have demonstrated that RvE1 plays a central role in musculoskeletal tissue repair. Turati et al. [13] reported that synovial RvE1 concentrations in individuals with anterior cruciate ligament injury were positively associated with intrinsic tissue repair potential. Similarly, Li et al. [14] showed in an experimental tendon–bone healing model that RvE1 enhances M2 macrophage polarization and type I collagen synthesis, thereby promoting structural integrity. These mechanisms are consistent with our findings, in which reduced RvE1 levels were associated with greater tendon retraction, suggesting impaired resolution-phase activity in chronic rotator cuff tears. Collectively, these data support the notion that RvE1 may serve as a key biochemical indicator of resolution capacity and tendon-specific healing dynamics.

In contrast, the absence of a significant association between RvD1 levels and tendon retraction or muscle fatty infiltration in our study indicates that RvD1 may exert a more limited influence within the chronic tendon microenvironment. Markworth et al. [17] demonstrated that RvD1 primarily supports myogenic repair by promoting myosatellite cell activation and early myofibre regeneration, with relatively weaker effects on tendon remodeling. Moreover, previous work has shown that RvD1’s target receptors, ALX/FPR2 and GPR32, are more abundantly expressed in monocytes and synovial macrophages compared with tendon cells [15]. This restricted receptor availability may explain the lack of structural correlation observed for RvD1. Together, these findings suggest that RvD1 functions predominantly during early inflammatory control, whereas RvE1 serves as the more relevant mediator in chronic tendon degeneration and repair. In addition to these mechanistic explanations related with RvE1 and RvD1 are derived from previous literature and are presented only to contextualize our clinical findings; the present study did not directly investigate molecular pathways.

Previous studies suggest that RvE1 may participate in preventing fibrotic remodeling and supporting tenocyte differentiation; these mechanisms were not examined directly in our study but provide relevant biological context [15,16]. The M2 macrophage phenotype associated with the RvE1–ChemR23 axis has been described to increase anti-inflammatory cytokines and suppress pro-inflammatory mediators; however, macrophage phenotypes were not measured in the present study [20]. This cellular reprogramming prevents prolongation of the resolution phase and inadequate tissue remodeling. Therefore, the low RvE1 levels observed in our study can be interpreted as a clinical reflection of dysfunction in the inflammation resolution process. Considering these detailed mechanisms, our findings suggest that RvE1 may be a biochemical indicator of inflammation resolution capacity in patients with rotator cuff tears, while RvD1 may play a regulatory role primarily in muscle regeneration and late healing phases. This difference suggests that RvE1 may hold potential as a diagnostic and prognostic biomarker, though further mechanistic and longitudinal studies are needed.

In our study, the significant differences in RvE1 levels in tendon pathologies indicate that this molecule is a dominant mediator in tissue-specific degradation dynamics. The literature reports that RvE1 balances the duration and intensity of the inflammatory response, particularly via the ChemR23–BLT1 axis [5,20]. In contrast, although RvD1 triggers similar signaling pathways via ALX/FPR2–GPR32 receptor activation, it is emphasized that the response amplitude at the tenocyte level is more limited [21,22] demonstrated that RvE1 significantly reduced COX-2 and IL-1β expression in rotator cuff disease tissues, whereas RvD1 did not produce a statistically significant reduction in the same tissues. Similarly, in an experimental model by [23] (2024), RvE1 administration increased the macrophage M2 polarization rate by 45%, while RvD1 produced an 18% increase under the same conditions. These findings support that RvE1 possesses a stronger anti-inflammatory reprogramming capacity, particularly at the tendon–bone interface. The studies by [24] (2019) and [25] (2024) indicated that RvE1 upregulates ChemR23 receptor expression in both myoblasts and tendon cells; in contrast, the target receptors of RvD1, ALX/FPR2 and GPR32, are predominantly concentrated in monocytes and synovial macrophages. This difference in receptor distribution may explain RvE1’s capacity to interact directly with tendon cells. Furthermore Dakin et al. (2014) [26] demonstrated that ChemR23 expression correlates with the reparative macrophage phenotype in chronic tendinopathies, confirming that the signaling target of RvE1 plays a critical role in tendon repair. On the other hand, it has been suggested that RvD1 plays a more active role in the early phase of the inflammatory response but is not as effective as RvE1 in creating a regenerative microenvironment. Markworth et al. (2020) [17,27,28] reported that RvD1 can support M2 polarization in muscle tissue, though this effect appears transient in tendon models; these mechanisms were not investigated in the present study. This difference indicates that RvE1 and RvD1 have different degradation profiles depending on timing and tissue context. In conclusion, consistent with the literature data, our findings show that RvE1 activates the ChemR23-mediated dissolution response in the tendon repair process in a more potent, sustainable, and cell-type-specific manner, while RvD1 provides a more limited and transient contribution. This difference highlights that RvE1 should be prioritized for evaluation as a therapeutic biomarker or target molecule for tendon regeneration.

**Figure 2:**
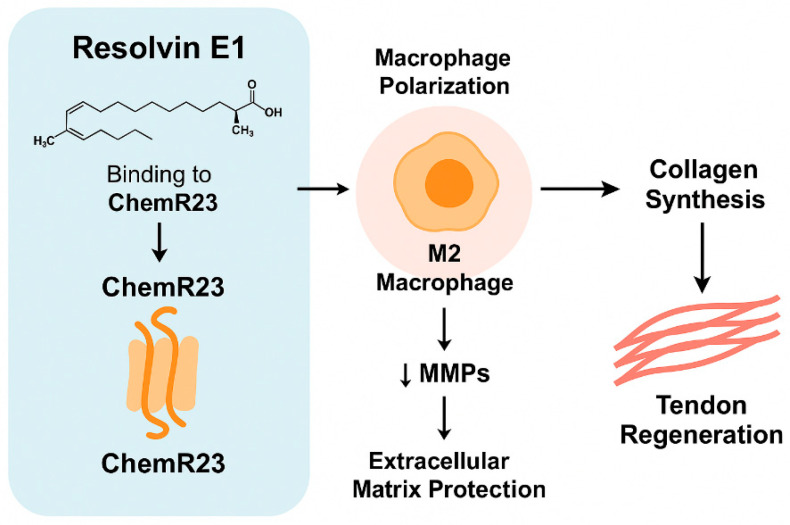
Effects of the RvE1–ChemR23 axis on tendon regeneration.

RvE1 is one of the specific lipid mediators that regulate the resolution phase of inflammation and modulates the duration and severity of the inflammatory response by acting through the ChemR23 receptor. The binding of RvE1 to ChemR23 suppresses the NF-κB and p38 MAPK signaling pathways, thereby reducing the expression of TNF-α, IL-1β, and COX-2 [20]. It also creates an anti-inflammatory microenvironment by increasing IL-10 and TGF-β3 production [16]. This dual effect allows the processes of tenocyte proliferation, ECM repair, and maintenance of mechanical integrity to commence upon resolution of inflammation [14,15]. High ChemR23 expression in tendon tissue, as reported in earlier studies, may enable RvE1 to influence tenocytes and M2 macrophages, although receptor expression was not assessed in our participants. This interaction activates ERK1/2 and Akt signaling, increasing COL1A1 and tenascin-C synthesis, thereby supporting tissue regeneration [23]. Furthermore, RvE1 has been reported to influence fibrotic remodeling by modulating MMP-9 and TIMP-1 activity; these pathways were not evaluated in our study [21]. These mechanistic explanations related with RvE1 and RvD1 are derived from previous literature and are presented only to contextualize our clinical findings; the present study did not directly investigate molecular pathways. These findings suggest that RvE1 is a potential therapeutic biomarker for tendon regeneration (Figure 2).

**Figure 3:**
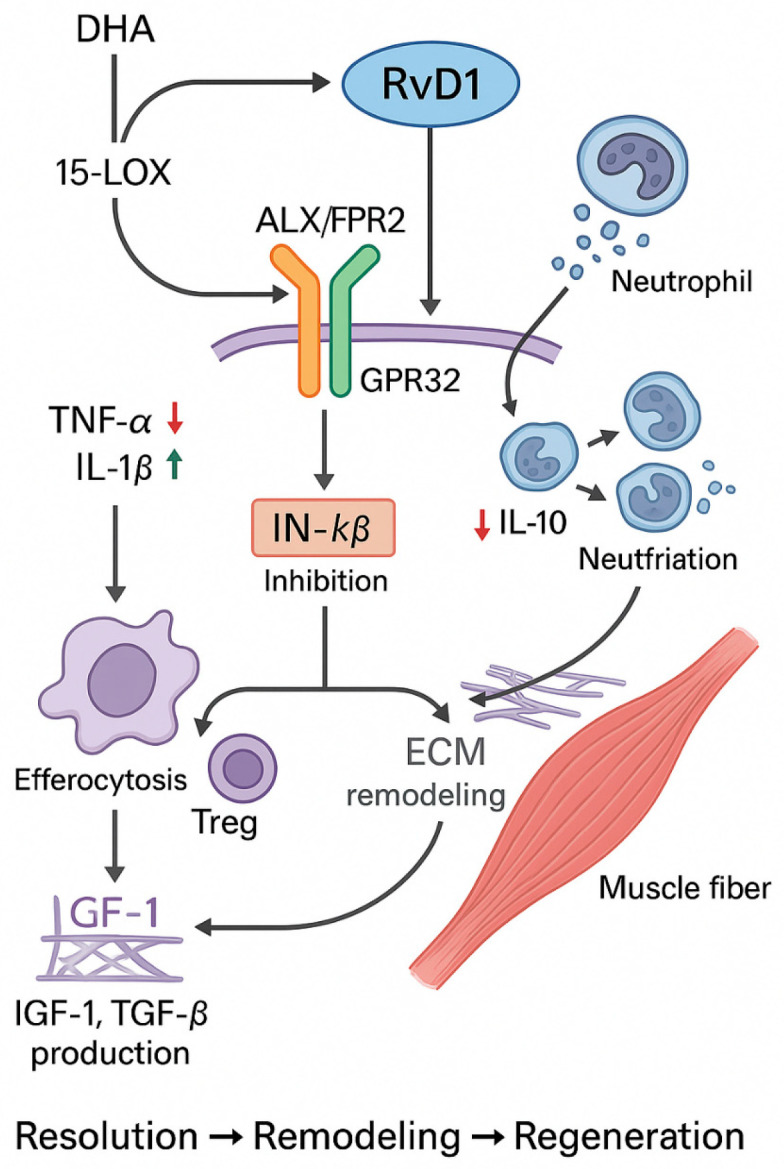
RvD1–ALX/FPR2-mediated limited dissolution response.

RvD1 is an important lipid mediator in the resolution of inflammation, but its effect in the tendon microenvironment is limited due to its dependence on ALX/FPR2–GPR32 receptors [6,7] (Figure 3). [17] reported that RvD1 supports M2 macrophage activation in muscle tissue, but this effect is transient and weak in tendon tissue. Low FPR2 receptor expression reduces the dissolution effect of RvD1 in tendon tissues, while the ChemR23-mediated RvE1 response becomes dominant [29]. This suggests that RvD1 is more effective in the early phase of inflammation, whereas RvE1 manages the repair process in the late phase. Current study data show that RvE1 provides the fundamental biological link between inflammation resolution and tendon regeneration. In contrast, due to the limited receptor availability and short duration of action in tendon tissue, RvD1 contributes to the resolution process at a secondary level. This difference supports the evaluation of RvE1 as a primary therapeutic target and potential biomarker in tendon pathologies [20,21]

Along with all these findings, this study has certain limitations. Firstly, the sample size is relatively small this may limit the generalizability of the results. Only serum RvE1 and RvD1 levels were analyzed; tissue-level expression, receptor localization (e.g., ChemR23, ALX/FPR2), or downstream signaling pathways were not evaluated. The patient group was older than the controls, and age showed a significant negative correlation with both RvE1 and RvD1 levels. Therefore, age represents a potential confounder that may partially influence circulating resolvin concentrations. Then, the study has a cross-sectional design; therefore, no conclusions can be drawn regarding the causal roles of resolvins in tendon healing or degeneration. Finally, participants’ dietary omega-3 intake which is known to influence resolvin biosynthesis was not assessed, and therefore represents a potential confounding factor. Systemic inflammatory status and medication use were also not quantitatively controlled. Nevertheless, this study provides an important starting point for future translational research by demonstrating the relationship between lipid mediators involved in the resolution phase of inflammation and the severity of tendon retraction.

The present findings suggest several practical implications for clinical management of rotator cuff tears. The significant association between reduced RvE1 levels and tendon retraction severity indicates that RvE1 may serve as a supportive biochemical marker in preoperative staging, particularly in estimating the chronicity and structural complexity of rotator cuff pathology. Monitoring RvE1 levels could potentially help identify patients at risk of advanced tendon degeneration, guide surgical timing, and support personalized rehabilitation strategies aimed at improving inflammation-resolution capacity. Although further prospective studies are needed, RvE1 may hold translational value as an accessible serum biomarker in shoulder injury assessment.

## 5. Conclusions

In this study, reduced serum RvE1 levels rather than RvD1 were associated with increased tendon retraction severity in patients with full-thickness rotator cuff tears. This finding suggests that impaired inflammation-resolution capacity may contribute specifically to tendon structural deterioration rather than to muscle fatty degeneration. As a result, RvE1 may represent a tendon-focused biochemical indicator reflecting the chronicity and degenerative progression of rotator cuff pathology. Further longitudinal and mechanistic studies are warranted to validate its potential as a clinically useful biomarker.

## Figures and Tables

**Table 1:** Demographic and clinical characteristics of participants.

	Patient Group (*n* = 35)	Healthy Group (*n* = 35)
Age (years, Mean ± SD)	63.1 ± 10.4	52.3 ± 8.8
Gender (Female/Male)	22/13	20/15
Affected Side	Right 24 (68.6%)/Left 11 (31.4%)	—

**Table 2:** Resolvin E1 (RvE1) and Resolvin D1 (RvD1) levels in patient and healthy groups.

	Biomarker	Patient	Healthy	t (Welch)	*p* Value	Effect Size (Hedges)
1	RvE1 (pg/mL)	82.18 ± 12.87	142.37 ± 97.05	−3.64	0.0009	−0.86
2	RvD1 (pg/mL)	81.63 ± 14.73	144.54 ± 8.55	−3.73	0.0007	−0.88

**Table 3:** Correlation between Serum Resolvin Levels and Morphological Scores.

Biomarker	Morphological Parameter	Spearman Rho (ρ)	*p*-Value
RvE1	Goutallier	0.19	0.27
RvE1	Patte	0.37	0.027
RvD1	Goutallier	0.13	0.46
RvD1	Patte	0.27	0.12

## Data Availability

The raw data supporting the conclusions of this article will be made available by the authors on request.
